# Facial sporotrichosis: case series, literature review and new insights about their clinical classification

**DOI:** 10.3389/fmed.2026.1780905

**Published:** 2026-04-13

**Authors:** Gaobo Ruan, Xiujiao Xia

**Affiliations:** Department of Dermatology, Hangzhou Third People's Hospital, Hangzhou Third Hospital Affiliated to Zhejiang Chinese Medical University, Hangzhou, China

**Keywords:** analysis, clinical classification, face, lymphocutaneous type, sporotrichosis

## Abstract

**Background:**

Cutaneous sporotrichosis is primarily categorized into lymphocutaneous (LC) and fixed (F) types. Sporotrichosis affecting the extremities typically manifests as a linear lymphangitic pattern, whereas facial sporotrichosis often exhibits atypical clinical features, thereby making it difficult to be classified.

**Objective:**

This study aims to analyze the distribution patterns of facial sporotrichosis and proposes novel insights into the clinical classification of atypical facial sporotrichosis based on facial lymphatic drainage patterns.

**Methods:**

This study presents a case series of seven cases with facial sporotrichosis and systematically reviews the relevant literature. All cases were evaluated for lesion distribution patterns (multifocal vs. localized) and linear arrangement (yes vs. no) according to our predefined criteria for classification.

**Results:**

Among our seven cases, six presented with multifocal distributions while one exhibited localized distributions. A systematic literature search identified 53 articles encompassing 84 facial sporotrichosis cases. Multifocal lesions predominated, occurring in 64 cases (76.2%). Among the 64 cases of facial sporotrichosis with multifocal lesions, 47 exhibited a non-linear pattern of lesion arrangement. This distribution comprised 26 of the 30 F-type cases, 7 of the 19 LC-type cases, and 14 of the 15 cases that were not definitively classified.

**Conclusion:**

Currently, the clinical classification of facial sporotrichosis presenting with multifocal and non-linear lesions remains unclear. Based on an analysis of the distribution patterns of these lesions and incorporating evaluation of facial lymphatic drainage characteristics, we propose that such multifocal, non-linear manifestations could be considered the LC type.

## 1 Introduction

Sporotrichosis is a subacute or chronic implantation (formerly subcutaneous) fungal infection, related to dimorphic fungi that fall within the *Sporothrix schenckii* complex. It is distributed worldwide, with a higher prevalence in tropical and subtropical regions, and is recognized as a neglected infectious disease. Traditionally, the clinical manifestations of sporotrichosis are classified into two main categories: cutaneous and extracutaneous. The cutaneous form is further subdivided into three subtypes: fixed cutaneous sporotrichosis (F), lymphocutaneous sporotrichosis (LC), and disseminated cutaneous sporotrichosis (1). In addition to this conventional classification, some scholars have proposed additional classificatory terms for cutaneous sporotrichosis, such as “multiple inoculation type” and “special type” (2, 3). Sporotrichosis affecting the extremities typically manifests as either a LC type with linear arrangement or F type, whereas facial sporotrichosis often presents atypical clinical manifestations that complicate clinical classification and frequently lead to misdiagnosis (4–8).

This study presents seven cases of facial sporotrichosis and conducts a systematic review of the relevant literature. By analyzing the distribution patterns of facial lesions from both our cases and those reported in the literature, we propose novel insights that aim to provide a new perspective on the clinical classification of atypical facial sporotrichosis.

## 2 Clinical case series

**Figure 1:**
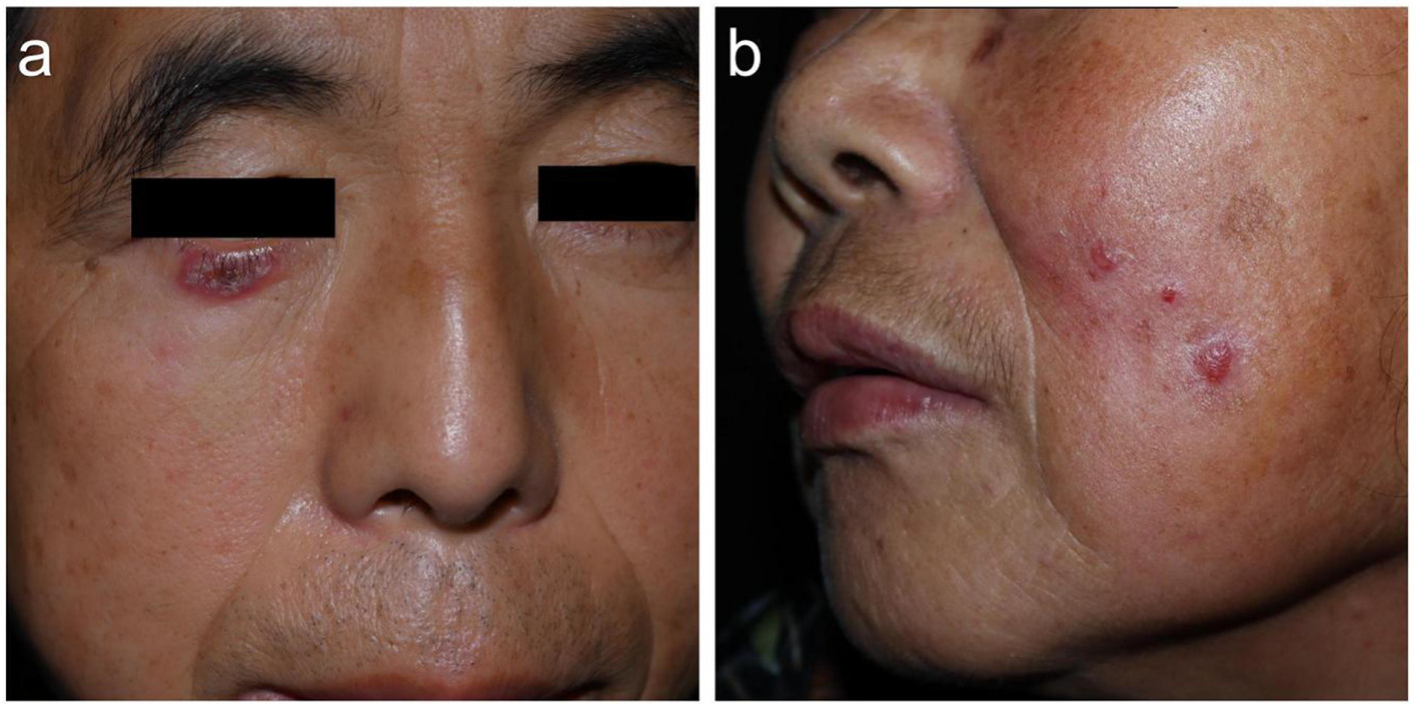
Facial sporotrichosis: presented with localized lesion in Case 1 **(a)**, and multifocal lesions in linear arrangement in Case 2 **(b)**.

**Figure 2:**
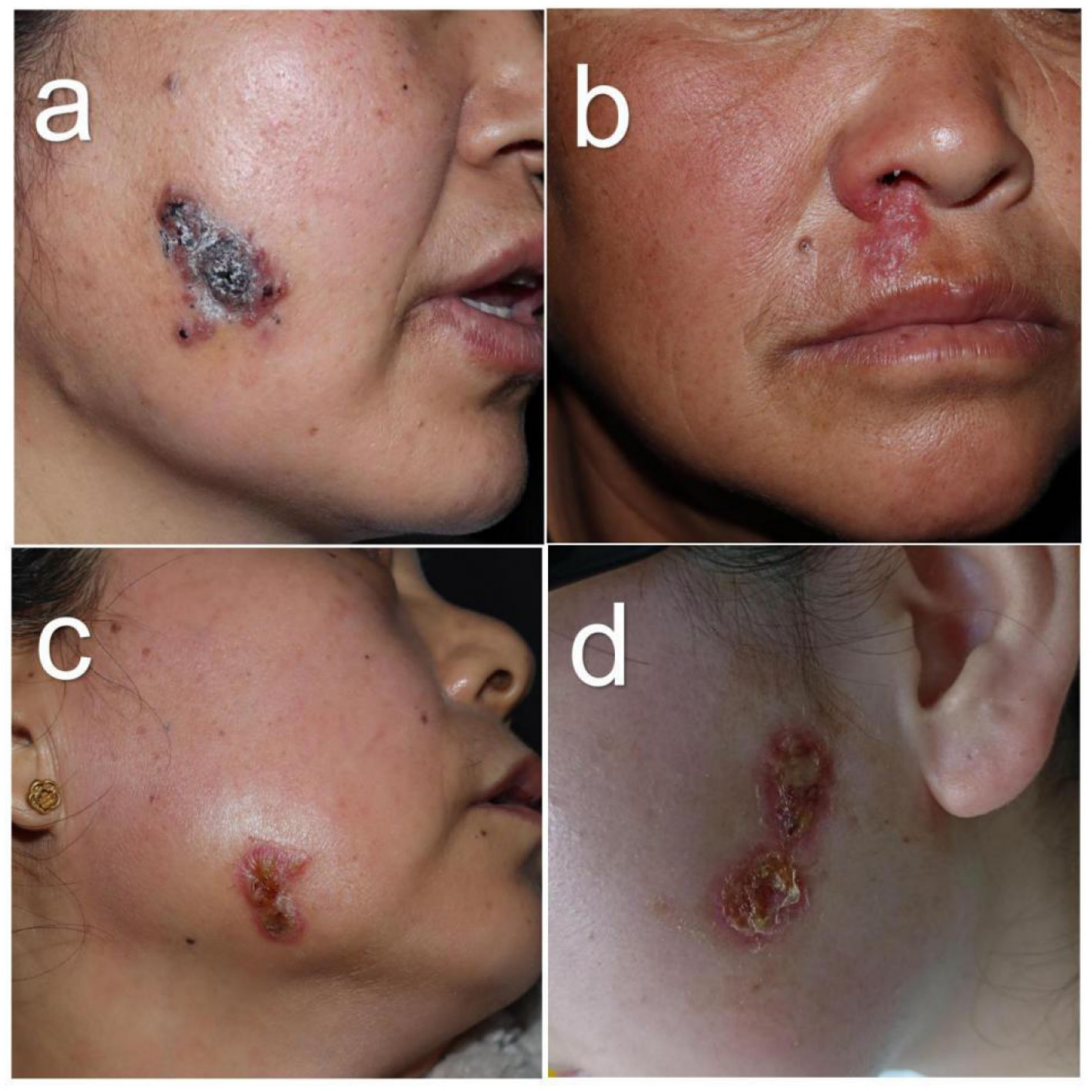
Facial sporotrichosis with multifocal lesions in non-linear arrangement: presented with satellite lesions in Case 3 **(a)**, and confluent lesions with indistinct borders in Case 4 **(b)**, Case 5 **(c)**, and Case 6 **(d)**.

**Figure 3:**
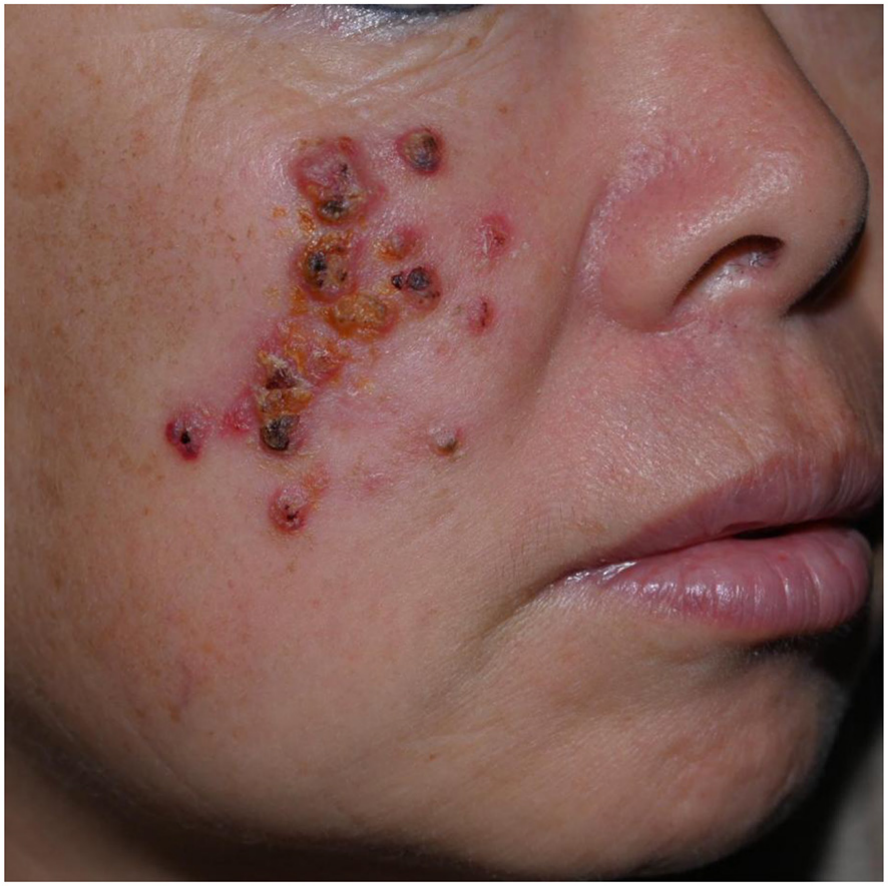
Facial sporotrichosis with lesions in non-linear arrangement: presented with scattered multifocal lesions in Case 7.

**Figure 4:**
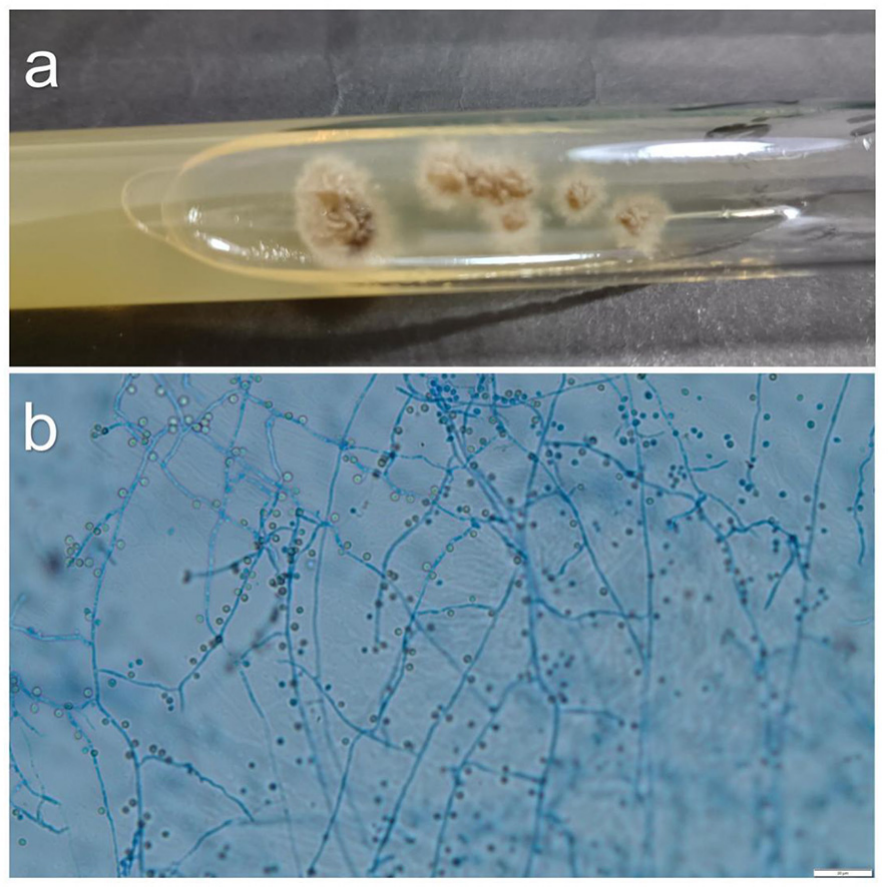
*S. globosa* colonies on SDA at 25°C on day 9 **(a)**; Slide culture of *S. globosa* on potato dextrose agar (PDA) at 25°C on day 7 **(b)**, lactic acid cotton blue staining; × 400).

Between January 2021 and December 2024, a total of 15 cases of facial sporotrichosis were diagnosed in the Department of Dermatology at Hangzhou Third People's Hospital. Among these, 8 cases have been reported in other publications (9–11). This retrospective analysis was performed on the remaining 7. Photographs of the patients were taken (Figures 1–3), and demographic information along with medical history were documented. Purulent secretions or biopsy tissues were collected for mycological examination. Specimens were inoculated onto Sabouraud Dextrose Agar (SDA) slants containing 50 μg/ml chloramphenicol and incubated at 25 ^°°^C for 10 days. Isolates were initially identified to genus level based on colonial and microscopic morphological characteristics (Figures 4a, b). Species-level identification was confirmed by sequencing of the internal transcribed spacer (ITS) or calmodulin (CAL) gene regions. All isolates obtained from the seven patients exhibited identical macroscopic and microscopic morphological characteristics *in vitro*. Furthermore, molecular identification confirmed that both our 7 new cases and the 8 previously published cases from our center were caused by *Sporothrix globosa*.

## 3 Methods

### 3.1 Ethics statement

All the methods were performed in accordance with the relevant approved guidelines, regulations and declaration of Helsinki. The study was approved by the ethics committee of Hangzhou Third People's Hospital (Approval No.: 2025KA177).

### 3.2 Literature search

A systematic literature search for cases of facial sporotrichosis was conducted in March 2025 using the National Library of Medicine (NLM) via PubMed, Scopus, and Web of Science. The search was limited to articles published in English and employed the following keywords: (“Sporotrichosis” OR “*Sporothrix*” OR “fixed cutaneous sporotrichosis” OR “lymphocutaneous sporotrichosis”) AND (“Facial” OR “Face” OR “Palpebral” OR “Eyelid” OR “Case Report”). We thoroughly reviewed the selected reference literature on an individual basis. Only studies containing clinical images sufficient for morphological analysis were included.

### 3.3 Evaluation methodology

To analyze the rash distribution patterns in facial sporotrichosis, lesions were categorized as either multifocal or localized. Localized distribution was defined as a single, isolated lesion confined to the face, Multifocal distribution was defined as the presence of two or more discrete or confluent lesions on the face. Cases with multifocal distribution patterns were further assessed to determine whether lesions exhibited linear or non-linear arrangements. Linear arrangement was defined as two or more lesions distributed continuously in a linear pattern. Non-linear arrangement was defined as two or more lesions distributed discretely, randomly, or in clusters without a linear trajectory. This study focuses on the re-evaluation of the clinical classification characterized by multifocal lesions. Both the categorization of distribution patterns and the assessment of linear arrangements were independently performed by two dermatologists. Any discrepancies between the two evaluators were resolved through mutual discussion.

## 4 Results

Among our seven cases, six presented with multifocal lesions and one with a localized lesion. Of the six cases with multifocal lesions, five showed a non-linear arrangement, while only one showed a linear arrangement (Table 1). To place our center's findings into a broader context, a systematic literature search was conducted. We identified 53 articles reporting 84 facial sporotrichosis cases, which included the 8 cases from our previously published study mentioned above. We reassessed 84 cases based on predefined classification criteria. Multifocal lesions predominated, accounting for 64 cases (76.2%), whereas localized lesions were identified in 20 cases (23.8%) (9) (Figures 6A, B) (12–16) (Cases) (3–5, 9, 11–13, 17–19) (Case 2); (13, 20) (Figures 1A–C). Among the 64 cases with multifocal lesions, 30 cases (46.9%) and 19 cases (29.7%) were originally classified in the literature as F-type and LC-type, respectively, with the remaining 15 cases (23.4%) being unclassified. When analyzing the arrangement patterns within these subtypes, a predominance of non-linear arrangement was observed across the F-type and unclassified cases, in contrast to the LC-type. Among the 30 F-type cases, non-linear arrangement predominated in 26 cases (86.7%) (4) (Cases 4, 5) (5, 6, 9) (Figures 6D–F) (16) (Cases 1, 8, 14, 15); (21–23); (24) (Cases 1–3) (25–30) (Cases 1, 2) (31, 32), whereas linear arrangement was observed in only 4 cases (13.3%) (9) (Figure 6C) (33–35). Similarly, among the 15 unclassified cases, non-linear arrangement was predominant in 14 cases (93.3%) (10, 11, 14, 36–46), with linear arrangement noted in 1 case (6.7%) (19) (Case 1). In contrast, among the 19 LC-type cases, linear arrangement was present in 12 cases (63.2%) (4) (Cases 1, 3) (16) (Cases 2, 10) (20) (Figures 1D, E) (45, 47–51), while non-linear arrangement was observed in 7 cases (36.8%) (4) (Case 2) (8, 16) (Cases 6, 7) (52–54) (Case 1). In summary, among the 64 cases of facial sporotrichosis with multifocal lesions, 47 exhibited a non-linear pattern of lesion arrangement. This distribution comprised 26 of the 30 F-type cases, 7 of the 19 LC-type cases, and 14 of the 15 cases that were not definitively classified (Table 2). In our cohort of six patients exhibiting multifocal manifestations, only one demonstrated a linear distribution. By contrast, among the 64 cases reported in the literature, 17 (26.6%) exhibited this pattern. It is worth noting that none of the cases included in this study involved a clear history of multifocal traumatic inoculation.

**Table 1:** Clinical characteristics for the 7 patients with facial sporotrichosis.

Case	Sex	Age	Disease duration (month)	Clinical classification	Distribution pattern	Linear arrangement (yes vs. No)	GenBank accession No.	Comorbidities	Treatment	Outcome
1	male	57	12	F	localized	—	PX634177	—	ITR/3months	Recovered
2	female	68	6	LC	Multifocal	Yes	PX633637	Hypertension; diabetes mellitus	ITR/3months	Recovered
3	female	48	3	LC	Multifocal	No	PX642400	**—**	ITR/3months	Recovered
4	female	59	6	LC	Multifocal	No	PX642407	Diabetes mellitus	ITR/3months	Recovered
5	female	48	8	LC	Multifocal	No	PX634155	Hepatitis C	ITR/3months	Recovered
6	female	24	12	LC	Multifocal	No	PX642401	**—**	ITR/3months	Recovered
7	female	61	5	LC	Multifocal	No	PX642402	Hypertension	ITR/3months	Recovered

F, fixed sporotrichosis; LC, lymphocutaneous sporotrichosis; ITR, itraconazole.

**Table 2:** Clinical classifications and distribution pattern of facial sporotrichosis cases with multifocal lesions in the literature.

Distribution pattern	*n* (%)	Linear arrangements, *n* (%)	Non-linear arrangements, *n* (%)
**Multifocal cases**	**64 (100)**		
NC	15 (23.4)	1 (6.7)	14 (93.3)
F	30 (46.9)	4 (13.3)	26 (86.7)
LC	19 (29.7)	12 (63.2)	7 (36.8)

F, fixed sporotrichosis; LC, lymphocutaneous sporotrichosis; NC, not classified

## 5 Discussion

Sporotrichosis is a subacute/chronic fungal disease that is caused by the dimorphic fungus of the genus *Sporothrix*. This fungal disease can affect both humans and livestock (55). At present, the consensus is that the *Sporothrix schenckii* complex includes *S. schenckii sensu stricto, S. globosa, S. brasiliensis*, and *S. luriei* (56). *S. globosa* and *S. schenckii* are globally distributed, with the former serving as the primary pathogen for human sporotrichosis in China (57).

Sporotrichosis exhibits a predilection for exposed body sites, particularly the extremities and face (58–60). The infection typically manifests following compromised epidermal barrier integrity secondary to minor trauma. Upon dermal invasion, the pathogen undergoes a thermally dependent dimorphic transition from the mycelial phase to the yeast phase. Subsequently, it may either establish localized colonies in subcutaneous tissue, resulting in the F type, or spread centripetally via lymphatic channels—forming subcutaneous nodules along lymphatic tracts—leading to the LC type. More rarely, hematogenous spread occurs, manifesting as the disseminated cutaneous form (1). Orofino-Costa et al. (2) proposed an alternative clinical classification system for human sporotrichosis, in which the cutaneous form is categorized into LC, F, and multifocal inoculation types. The multifocal inoculation type, characterized by multiple polymorphic lesions at non-contiguous sites in the absence of systemic involvement, is considered the rarest manifestation of cutaneous sporotrichosis. Professor Li Fuqiu (3) classified cutaneous sporotrichosis into LC, F, special, and cutaneous disseminated forms. The special type primarily presents on the face and may clinically resemble acne, rosacea, or herpes zoster. It is evident that these classification systems have increasingly focused on these special clinical presentations characterized by non-linear distribution.

Among the 47 cases with multifocal lesions in a non-linear arrangement, the majority were originally classified as F-type (26/47, 55.3%). In our previously published studies on facial sporotrichosis (9–11), we also categorized such multifocal non-linearly arranged cases either as F-type or described them as “atypical facial sporotrichosis”. In fact, the defining feature of the LC type lies in whether the skin lesions disseminate along lymphatic drainage, rather than merely depending on the presence of a classic “linear distribution”.

**Figure 5:**
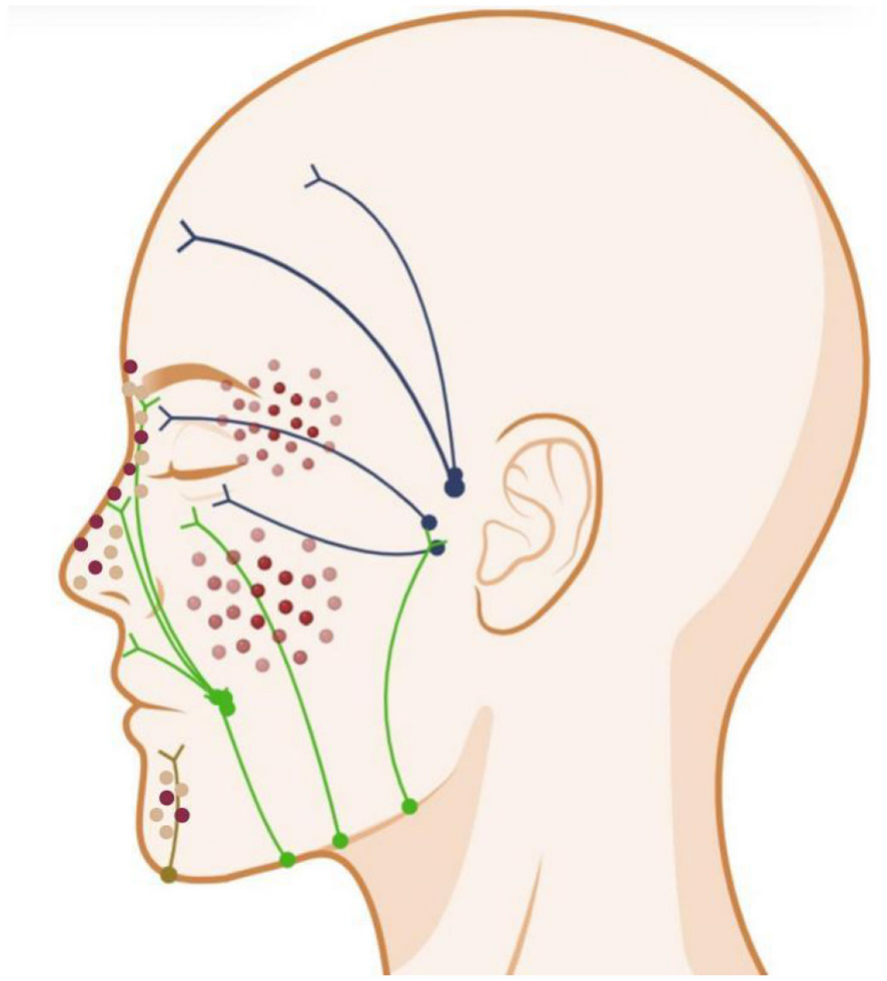
Schematic representation of facial lymphatic drainage territories. The facial lymphatic basins are color-coded as follows (Based on systematic anatomical principles in conjunction with Pan et al. (61).): Blue: Parotid nodal territory draining the temporal scalp, forehead, upper eyelids, lateral lower eyelids, and anterior auricle; Green: Submandibular basin receiving lymph from medial lower eyelids, cheek, nasal dorsum, upper/lower lips, and lateral mentum; Brown: Submental compartment collecting drainage from central lower lip, mentum, and submental cutaneous region. Created in BioRender. Ruan, G. (2026) https://BioRender.com/cddip2z.

Facial lymphatic vessels exhibit intricate anatomical complexity, traveling radially from medial to lateral with extensive branching and frequent anastomoses (61) (Figure 5). This configuration may facilitate pathogen dissemination through divergent lymphatic pathways, potentially serving as a critical determinant for the irregular distribution of cutaneous lesions in facial sporotrichosis compared to the typical linear lesions observed on the limbs.

You and Ran (52) reported a case of symmetrical sporotrichosis on the face, suggesting that these bilateral manifestations may be caused by diffusion of infection in the nose along the nasal (arising on both lateral external sides of the nose) and eyelid (arising on the inner canthus) branches of the facial lymphatic vessels. Arumugam et al. (62) reported a case of epigastric sporotrichosis, demonstrating a diagonal spread pattern toward the bilateral axillary lymph nodes. The authors attributed this distinctive pattern to the unique drainage pathways of abdominal wall lymphatics, noting that supraumbilical regions exhibit diagonal drainage toward axillary nodal basins.

While traditional classification defines the F-type as strictly localized and the LC-type as spreading linearly, this binary classification does not fully account for the unique facial lymphatic anatomy. The fundamental pathophysiology defining the LC-type is contiguous dissemination via the lymphatic system. Because facial lymphatics form a complex, web-like network with extensive radial branching and anastomoses (unlike the straightforward unidirectional pathways of the limbs), lymphatic spread on the face naturally manifests as clustered, satellite, or irregularly radiating lesions. Therefore, we propose that classifying such non-linear multifocal presentations as the F-type is inconsistent with the biological basis of lymphatic dissemination. Logically, these warrant reclassification as the LC-type, as the underlying mechanism remains lymphatic spread, merely modified by regional anatomy.

Based on the preceding analysis, we suggest that the classification criteria for facial sporotrichosis could be based on the number and distribution pattern of lesions. Specifically: (1) cases with a single lesion are categorized as F-type; (2) cases with ≥2 discrete lesions in a linear arrangement are categorized as LC-type; and (3) cases with ≥2 discrete lesions presenting as either satellite configurations, multifocal scattered distributions, or confluent lesions with indistinct borders are categorized as LC-type. The exclusion of a history of multiple traumatic inoculations is the basis for these classifications.

According to the above classification criteria, the seven facial sporotrichosis cases in our series were categorized as follows: Case 1 was the F-type, while Cases 2 to 7 were the LC- type. Within the LC group, Case 2 demonstrated a linear arrangement, while Cases 3 to 7 exhibited non-classic LC-type manifestations: specifically, Case 3 presented with satellite configurations, Cases 4–6 with confluent lesions with indistinct borders, and Case 7 with multifocal scattered distributions. We also compared the distribution patterns, lesion arrangement, and clinical classification between the cases from our series (*n* = 7) and those reported in the literature (*n* = 84) (Table 3). Both groups demonstrated a similarity in distribution patterns, with multifocal lesions being the predominant manifestation (85.7% vs. 76.2%). Furthermore, within the multifocal cases, non-linear arrangements were prevalent in both cohorts (83.3% vs. 73.4%). However, a significant discrepancy emerged regarding clinical classification. Among the multifocal cases with non-linear arrangement in the literature, the majority had originally been classified as F-type (26/30, 86.7%) or remained unclassified (14/15, 93.3%), whereas only a minority had been classified as LC-type (7/19, 36.8%). According to our criterion, all 47 cases with a non-linear distribution can be reclassified as the LC type. It is worth noting that seven of these cases were originally classified as the LC type in the source text, which is consistent with our criterion. This indicates that some scholars have already recognized that the classification of the LC type is not dependent on the clinical manifestation of linear distribution.

**Table 3:** Comparative analysis of distribution patterns, lesion arrangement, and clinical classification between our series and the literature.

Features	Our series (*n* = 7)	Literature (*n* = 84)
Distribution pattern, *n* (%)		
Localized	1 (14.3)	20 (23.8)
Multifocal	6 (85.7)	64 (76.2)
Arrangement in multifocal cases, *n* (%)	(*n* = 6)	(*n* = 64)
Non-linear arrangement	5 (83.3)	47 (73.4)
In our series: multifocal cases by our classification, *n* (%)	(*n* = 6)	—
Among LC-type (*n* = 6)	6 (100)	—
In the literature: non-linear cases by original classification, *n* (%)		
Among F-type (*n* = 30)	—	26 (86.7)
Among LC-type (*n* = 19)	—	7 (36.8)
Among NC (*n* = 15)	—	14 (93.3)

F, fixed sporotrichosis; LC, lymphocutaneous sporotrichosis; NC, not classified.

The present classification framework offers two key contributions. First, it provides a standardized, operationally feasible approach to categorizing lesion distribution patterns in facial sporotrichosis, thereby facilitating consistent clinical documentation and communication. Second, this framework enhances clinical recognition by clarifying that the defining feature of the LC-type is contiguous lymphatic dissemination, not the morphological presence of linear arrangement. Accordingly, clinicians should maintain a high index of suspicion for facial sporotrichosis when encountering indolent, non-healing, multifocal grouped, or satellite noduloulcerative lesions on the face—even in the absence of linear tracking—particularly when standard antibacterial or topical therapies have failed. Timely mycological culture and molecular identification remain essential for a definitive diagnosis.

## 6 Conclusions

This study describes seven cases of facial sporotrichosis and presents a systematic review of the relevant literature, underscoring the complexity of clinical classification in facial sporotrichosis. The lymphatic vessels of the face typically exhibit a mediolaterally complex radial branching pattern with frequent anastomoses, which may underlie the distinctive presentation of facial sporotrichosis as dendritic, irregularly radiating, or clustered lesions, as opposed to the classic linear arrangement. Based on the aforementioned observations, we propose that facial sporotrichosis presenting with multifocal, non-linear manifestations may be considered the LC type, unless there is a clear history of multifocal traumatic inoculation. This study may provide novel insights into classification criteria for atypical facial sporotrichosis, thereby aiding in clinical recognition and management.

### 6.1 Limitations

It is important to acknowledge several limitations of this study. First, the retrospective nature of both our case series and the literature review inherently introduces selection and reporting biases. Second, the relatively small sample size may limit the generalizability of our findings. Third, the proposed classification criteria lack direct objective validation; for instance, imaging techniques such as indocyanine green (ICG) lymphography were not utilized to definitively prove lymphatic tracking between the non-linear lesions. Consequently, the classification of such cases as LC-type remains inferential rather than directly demonstrated. Future large-scale, prospective studies incorporating advanced lymphatic imaging are required to validate this hypothesis.

## Data Availability

The original contributions presented in the study are included in the article/supplementary material, further inquiries can be directed to the corresponding authors.

## References

[1] Barros MB de Almeida Paes R Schubach AO. Sporothrix schenckii and Sporotrichosis. Clin Microbiol Rev. (2011) 24:633–54. doi: 10.1128/CMR.00007-11 21976602 PMC3194828

[2] Orofino-Costa R Freitas DFS Bernardes-Engemann AR Rodrigues AM Talhari C Ferraz CE . Human sporotrichosis: recommendations from the Brazilian Society of Dermatology for the clinical, diagnostic and therapeutic management. An Bras Dermatol. (2022) 97:757–77. doi: 10.1016/j.abd.2022.07.001 36155712 PMC9582924

[3] Zhao X Li P Yang J. Clinical presentation and histopathological characteristics of sporotrichosis. J Cosmet Dermatol. (2025) 24:e70059. doi: 10.1111/jocd.70059 40084684 PMC11907742

[4] Shi W Zheng Y Wang H Zhang R. Misdiagnosis of cutaneous facial sporotrichosis: an analysis of five cases. J Cosmet Dermatol. (2024) 23:3000–4. doi: 10.1111/jocd.16335 38654514

[5] Tang Q Zhou Y Chen Y Chen J Xiong X. Dynamic observation of atypical sporotrichosis before and after itraconazole treatment by dermoscopy. Clin Cosmet Investig Dermatol. (2023) 16:339–43. doi: 10.2147/CCID.S400302 36762257 PMC9904292

[6] Zhu P Yu J. Facial recurred fixed sporotrichosis. Mycopathologia. (2021) 186:313–4. doi: 10.1007/s11046-020-00516-y 33483861

[7] Hong YS Liu LF Hu HH. Sporotrichosis mimicking rosacea lesions: a case report. Clin Cosmet Investig Dermatol. (2024) 17:1033–6. doi: 10.2147/CCID.S461558 38737947 PMC11088399

[8] Sharma NL Mehta KI Mahajan VK Kanga AK Sharma VC Tegta GR. Cutaneous sporotrichosis of face: polymorphism and reactivation after intralesional triamcinolone. Indian J Dermatol Venereol Leprol. (2007) 73:188–90. doi: 10.4103/0378-6323.32745 17558054

[9] Zhu H Xia X Zhi H Shen H Lv W Sang B . Retrospective analysis of 71 patients with cutaneous sporotrichosis. Mycoses. (2023) 66:621–31. doi: 10.1111/myc.13588 37035906

[10] Ruan G Xia X. Atypical Facial Sporotrichosis. Mycopathologia. (2025) 190:44. doi: 10.1007/s11046-025-00953-7 40405048

[11] Xia X Zhi H Liu Z. Cutaneous Sporotrichosis Following a Brow Lift. Mycopathologia. (2023) 188:419-420. doi: 10.1007/s11046-023-00753-x 37284913

[12] Ma X Ma DL. Fixed Facial Sporotrichosis. Indian J Pediatr. (2025) 92:191–2. doi: 10.1007/s12098-024-05319-3 39480617

[13] Liu F Liu Y Yuan N Zhang X Cao M Dong J . Fixed Cutaneous Sporotrichosis Due to Sporothrix globosa. Clin Cosmet Investig Dermatol. (2021) 14:91–6. doi: 10.2147/CCID.S288259 33531824 PMC7846868

[14] Barraza LL Tolomelli JB Cunha CG Bernardes Filho F Towersey L Hay R . Facial Cutaneous Sporotrichosis in a Boy. J Emerg Med. (2019) 56:222–3. doi: 10.1016/j.jemermed.2018.10.031 30527560

[15] Mahajan VK Mehta KS Chauhan PS Gupta M Sharma R Rawat R. Fixed cutaneous sporotrichosis treated with topical amphotericin B in an immune suppressed patient. Med Mycol Case Rep. (2015) 7:23–5. doi: 10.1016/j.mmcr.2015.01.002 27330943 PMC4909862

[16] Song Y Yao L Zhong SX Tian YP Liu YY Li SS. Infant sporotrichosis in northeast China: a report of 15 cases. Int J Dermatol. (2011) 50:522–9. doi: 10.1111/j.1365-4632.2010.04724.x 21506965

[17] Tlougan BE Podjasek JO Patel SP Nguyen XH Hansen RC. Neonatal sporotrichosis. Pediatr Dermatol. (2009) 26:563–5. doi: 10.1111/j.1525-1470.2009.00986.x 19840311

[18] Guo K Wang S Wang Z Zhang L. Effective treatment using itraconazole combined with terbinafine in the treatment of nasal sporotrichosis: a case report. Medicine. (2019) 98:e17155. doi: 10.1097/MD.0000000000017155 31517860 PMC6750297

[19] Inokuma D Shibaki A Shimizu H. Two cases of cutaneous sporotrichosis incontinental/ microthermal climate zone: global warming alert? Clin Exp Dermatol. (2010) 35:668-9. doi: 10.1111/j.1365-2230.2010.03795.x 20184611

[20] Fan B Wang JF Zheng B Qi XZ Song JY Li GY. Clinical features of 10 cases of eyelid sporotrichosis in Jilin Province (Northeast China). Can J Ophthalmol. (2016) 51:297–301. doi: 10.1016/j.jcjo.2016.02.018 27521671

[21] Xiang YK Qu T. Image gallery: facial sporotrichosis. Br J Dermatol. (2018) 178:e275. doi: 10.1111/bjd.16358 29668090

[22] Song JG Song YB Yun SY Suh MK Ha GY Kim JR . Cutaneous Sporotrichosis Presenting as Clinical Feature of Facial Cellulitis in an Adult. Ann Dermatol. (2016) 28:507-8. . doi: 10.5021/ad.2016.28.4.507 27489440 PMC4969487

[23] Fulghum K Mollet T Nguyen J. What is your diagnosis? Fixed cutaneous sporotrichosis. Cutis. (2015) 96:218, 227-8. 26682291

[24] Zhang Y Pyla V. Nasal sporotrichosis in children. Int J Dermatol. (2014) 53:e342-3. doi: 10.1111/ijd.12312 24372190

[25] Zhang Y Pyla V. Palpebral sporotrichosis. Int J Dermatol. (2014) 53:e356–7. doi: 10.1111/ijd.12382 24372286

[26] Ramírez-Soto M Lizárraga-Trujillo J. Esporotricosis granulomatosa: presentación de dos casos inusuales [Granulomatous sporotrichosis: report of two unusual cases]. Rev Chilena Infectol. (2013) 30:548-53. doi: 10.4067/S0716-10182013000500013 24248171

[27] Lauermann F Lyra M Gaudio R. Sporotrichosis mimicking keratoacanthoma. Am J Trop Med Hyg. (2012) 86:741. doi: 10.4269/ajtmh.2012.11-0681 22556066 PMC3335672

[28] Ramesh V Singh A Pahwa M Capoor M. A recalcitrant facial plaque: fixed cutaneous sporotrichosis. Int J Dermatol. (2011) 50:367–8. doi: 10.1111/j.1365-4632.2009.04354.x 21342173

[29] Noguchi H Hiruma M Kawada A. Case report. Sporotrichosis successfully treated with itraconazole in Japan. Mycoses. (1999) 42:571–6. doi: 10.1046/j.1439-0507.1999.00484.x 10592704

[30] Prose NS Milburn PB Papayanopulos DM. Facial sporotrichosis in children. Pediatr Dermatol. (1986) 3:311–4. doi: 10.1111/j.1525-1470.1986.tb00531.x 3774649

[31] Dellatorre DL Lattanand A Buckley HR Urbach F. Fixed cutaneous sporotrichosis of the face. Successful treatment of a case review of the literature. J Am Acad Dermatol. (1982) 6:97–100. doi: 10.1016/S0190-9622(82)80204-1 7085959

[32] Pepper MC Rippon JW. Sporotrichosis presenting as facial cellulitis. JAMA. (1980) 243:2327–8. doi: 10.1001/jama.1980.03300480047026 7373802

[33] Taninratapat N Srisuttiyakorn C. Localized Cutaneous Sporotrichosis on Face in Healthy Thai Female. Mycopathologia. (2019) 184:539-42. . doi: 10.1007/s11046-019-00354-7 31309401

[34] Zhang Y Pyla V. Sweet's syndrome-like sporotrichosis. Int J Dermatol. (2014) 53:e324–5. doi: 10.1111/ijd.12424 24372350

[35] Khaitan BK Lakhanpal S Banerjee U Pandhi RK. Sporotrichosis in an unusual location–dermatologically and geographically. Indian J Pathol Microbiol. (1998) 41:461–3. 9866909

[36] Del Rio FV Rodrigues FT Melo RR Quirino RM Azulay-Abulafia L. Cutaneous Sporotrichosis of Face with Verrucous Lesions over Nose, Successfully Treated with a Combination of Itraconazole and Terbinafine: a case report. Indian Dermatol Online J. (2023) 14:276-78. doi: 10.4103/idoj.idoj_259_22 37089842 PMC10115338

[37] Robles-Tenorio A Rocha-Mendez LE Tarango-Martinez VM. Cryosurgery as adjuvant treatment for cutaneous sporotrichosis in two patients with diabetes mellitus type 2. Clin Exp Dermatol. (2023) 48:257–9. doi: 10.1093/ced/llac090 36763769

[38] Li L Zhang S Pradhan S Ran Y. Facial Sporotrichosis with Liver Cirrhosis Detected by Calcofluor White Treated with Itraconazole. Mycopathologia. (2021) 186:141–2. doi: 10.1007/s11046-020-00518-w 33392859

[39] Liang T Zhou Y Wan Z Li R Liu W. A case of sporotrichosis. Int J Infect Dis. (2020) 96:163-64. doi: 10.1016/j.ijid.2020.04.032 32305519

[40] Martins-Costa GM Bonamigo RR. Facial verrucous sporotrichosis in an adult during treatment for rosacea. Int J Dermatol. (2014) 53:e124–6. doi: 10.1111/j.1365-4632.2012.05688.x 23557535

[41] Williams BA Jennings TA Rushing EC Wirges M Russell BE. Sporotrichosis on the face of a 7-year-old boy following a bicycle accident. Pediatr Dermatol. (2013) 30:e246–7. doi: 10.1111/j.1525-1470.2011.01696.x 22299662

[42] Parekh PK Butler DF. What is your diagnosis? Periorbital granulomatous plaque. Pediatr Dermatol. (2011) 28:457–8. doi: 10.1111/j.1525-1470.2011.01499.x 21793885

[43] Bonifaz A Saúl A. Montes-de-Oca G, Mercadillo P. Superficial cutaneous sporotrichosis in specific anergic patient. Int J Dermatol. (1999) 38:700–3. doi: 10.1046/j.1365-4362.1999.00742.x 10517689

[44] Romig DA Voth DW Liu C. Facial sporotrichosis during pregnancy. A therapeutic dilemma. Arch Intern Med. (1972) 130:910–2. doi: 10.1001/archinte.1972.03650060100018 5082472

[45] Gameiro Filho AR Estacia CT Gameiro RR de Mattos Fonseca Vieira L Socci da. Costa D. Ocular and cutaneous sporotrichosis. Am J Ophthalmol Case Rep. (2020) 20:100885. doi: 10.1016/j.ajoc.2020.100885 32875162 PMC7452058

[46] Tochigi M Ochiai T Mekata C Nishiyama H Anzawa K Kawasaki M. Sporotrichosis of the face by autoinoculation in a patient undergoing tacrolimus treatment. J Dermatol. (2012) 39:796–8. doi: 10.1111/j.1346-8138.2011.01420.x 22168566

[47] Li X Ma DL. Linear nodules on the face: a case of facial lymphocutaneous sporotrichosis. Br J Dermatol. (2025) 192:ljaf010. 39761691 10.1093/bjd/ljaf010

[48] Verma S Verma G Rattan R. Lymphocutaneous Sporotrichosis of Face with Verrucous Lesions: a case report. Indian Dermatol Online J. (2019) 10:303–6. doi: 10.4103/idoj.IDOJ_272_18 31149578 PMC6536070

[49] Ramírez Soto MC Andagua-Castro J Lizárraga-Trujillo J. Palpebral sporotrichosis in a 6-year-old child. Int J Dermatol. (2016) 55:e625–6. doi: 10.1111/ijd.13383 27496011

[50] Zhang Y Pyla V. Cancer-like lesions in a patient with sporotrichosis. Int J Dermatol. (2014) 53:e311–2. doi: 10.1111/ijd.12151 24262023

[51] Iyengar SS Khan JA Brusco M FitzSimmons CJ. Cutaneous Sporothrix schenckii of the human eyelid. Ophthalmic Plast Reconstr Surg. (2010) 26:305–6. doi: 10.1097/IOP.0b013e3181c15c1f 20551856

[52] You Z Ran Y. Symmetrical sporotrichosis on the face: pathogen spread from the nose along both sides of facial lymphatic vessels? Br J Dermatol. (2023) 189:784–5. doi: 10.1093/bjd/ljad307 37610858

[53] Sharma NL Mahajan VK Verma N Thakur S. Cutaneous sporotrichosis: an unusual clinico-pathologic and therapeutic presentation. Mycoses. (2003) 46:515–8. doi: 10.1046/j.0933-7407.2003.00934.x 14641627

[54] Ramírez-Oliveros JF Casz Schechtman R de Vries HJ Lora L Cardoso Arinelli A da Costa Nery JA . Ocular adnexal sporotrichosis: a case series. JAAD Case Rep. (2021) 13:52–6. doi: 10.1016/j.jdcr.2021.04.019 34150968 PMC8190125

[55] Conceição-Silva F Morgado FN. Immunopathogenesis of Human Sporotrichosis: What We Already Know. J Fungi. (2018) 4:89. doi: 10.3390/jof4030089 30065160 PMC6162489

[56] Rodrigues AM Della Terra PP Gremião ID Pereira SA Orofino-Costa R de Camargo ZP. The threat of emerging and re-emerging pathogenic Sporothrix species. Mycopathologia. (2020) 185:813–42. doi: 10.1007/s11046-020-00425-0 32052359

[57] Yu X Wan Z Zhang Z Li F Li R Liu X. Phenotypic and molecular identification of Sporothrix isolates of clinical origin in Northeast China. Mycopathologia. (2013) 176:67–74. doi: 10.1007/s11046-013-9668-6 23771481 PMC3731519

[58] Lv S Hu X Liu Z Lin Y Wu H Li F. Clinical Epidemiology of Sporotrichosis in Jilin Province, China (1990-2019): A Series of 4969 Cases. Infect Drug Resist. (2022) 15:1753-65. doi: 10.2147/IDR.S354380 35431560 PMC9012317

[59] Ramírez Soto MC. Sporotrichosis: The Story of an Endemic Region in Peru over 28 Years (1985 to 2012). PLoS ONE. (2015) 10:e0127924. doi: 10.1371/journal.pone.0127924 26030742 PMC4452310

[60] Arenas R Sánchez-Cardenas CD Ramirez-Hobak L Ruíz Arriaga LF Vega Memije ME. Sporotrichosis: From KOH to Molecular Biology. J Fungi. (2018) 4:62. doi: 10.3390/jof4020062 29882883 PMC6023365

[61] Pan WR Le Roux CM Briggs CA. Variations in the lymphatic drainage pattern of the head and neck: further anatomic studies and clinical implications. Plast Reconstr Surg. (2011) 127:611–20. doi: 10.1097/PRS.0b013e3181fed511 21285766

[62] Arumugam M Leelavathi M Harun NL Jamil A. Lymphocutaneous sporotrichosis of the abdominal wall: a lesson in lymphatic drainage. Med J Malaysia. (2021) 76:583–4. 34305124

